# Relationship of inflammatory and metabolic parameters in adolescents with PCOS: BMI matched case-control study

**DOI:** 10.20945/2359-3997000000497

**Published:** 2022-06-02

**Authors:** Aysun Tekeli Taşkömür, Özlem Erten

**Affiliations:** 1 Amasya University Faculty of Medicine Department of Obstetrics and Gynecology Amasya Turkey Amasya University, Faculty of Medicine, Department of Obstetrics and Gynecology, Amasya, Turkey; 2 Kütahya Health Sciences University Faculty of Medicine Department of Gynecology and Obstetrics Kütahya Turkey Kütahya Health Sciences University, Faculty of Medicine, Department of Gynecology and Obstetrics, Kütahya, Turkey

**Keywords:** Adolescent, polycystic ovary syndrome, cystatin C, high-sensitivity C-reactive protein, neutrophil-lymphocyte ratio, platelet-lymphocyte ratio

## Abstract

**Objective:**

Polycystic ovary syndrome (PCOS) begins in adolescence and has cardiovascular and metabolic components in later years. Cystatin C and high-sensitivity C-reactive protein (hs-CRP) levels and neutrophil-lymphocyte and platelet-lymphocyte ratios are associated with metabolic and inflammatory events. Here, we evaluated inflammatory and metabolic parameters in normal and overweight adolescents with PCOS.

**Materials and methods:**

This prospective case-control study enrolled 90 adolescents with PCOS and 100 matched by age and BMI healthy adolescents classified as either normal weight (NW) and overweight (OW). Groups were compared based on inflammatory and metabolic parameters (serum cystatin C, hs-CRP, neutrophil-lymphocyte ratio (NLR), platelet-lymphocyte ratio (PLR), lipids, fasting blood glucose-insulin (FBG-FI), HOMA-IR levels, waist circumference [WC], and waist-hip ratio [WHR]). The relationship between the parameters were compared and predictive abilities were evaluated.

**Results:**

Cystatin C, hs-CRP, NLR, triglyceride (TG), FBG-FI, HOMA-IR, WC, and WHR were significantly higher in those with PCOS. The NW PCOS group had significantly higher TG, cystatin C, hs-CRP, and NLR versus OW controls. The highest HOMA-IR values were observed in OW PCOS (*p* < .05). Cystatin C and hs-CRP sensitivity and specificity were significant (*p* < 0.05). Cystatin C and hs-CRP were positively correlated with other metabolic parameters.

**Conclusion:**

Independent of BMI, inflammatory and metabolic parameters are significantly higher in adolescents with PCOS compared to controls and even worse in those who are also OW. Therefore, adolescents with PCOS should be encouraged to maintain healthy lifestyles and weights to avoid metabolic risks. Hs-CRP and cystatin C could be promising markers to predictive of future metabolic risks.

## INTRODUCTION

Polycystic ovary syndrome (PCOS) is characterized by chronic anovulation, hyperandrogenism, and ovaries with a polycystic appearance. PCOS is an endocrinopathy with systemic, inflammatory, and lifestyle effects (1). It can occur in the adolescent period and is the most common endocrine system disorder in women of reproductive age affecting 4%-8% of women worldwide (2). Its prevalence in adolescents is approximately 1.14% (3). The clinical presentation of PCOS in adolescents is usually menstrual cycle disorder and hirsutism (4).

PCOS in adolescence is associated with an increased risk of metabolic syndrome, type 2 diabetes mellitus, and diseases of the cardiovascular system (5). Metabolic syndrome is a disorder with glucose abnormalities, obesity, hypertension, hyperlipidemia, and insulin resistance. It is present in approximately 25% of adolescents with PCOS (6). Obesity and insulin resistance are frequently seen in PCOS and also trigger a chronic inflammatory process (7). For patients with PCOS, finding markers for future metabolic risks and their severity is important in preventing long-term effects.

Cystatin C is an extracellular cysteine protease inhibitor. Cysteine protease activity may play a role in many conditions such as inflammation and tumor metastasis and is a novel cardiometabolic risk marker for women with PCOS (8,9).

Hs-CRP is synthesized in the liver and is the most sensitive acute-phase reactant. Synthesis is stimulated by interleukin-1, interleukin-6, and tumor necrosis factor. Studies have shown a relationship between hs-CRP and inflammatory processes such as atherosclerosis, cancer progression and type 2 diabetes mellitus. Hs-CRP is elevated in women with PCOS (10,11).

Neutrophils are defense precursor cells of the immune system originating from the bone marrow. Platelets and neutrophils increase cytokines’ secretion at the onset of inflammation, and cytokines contribute to inflammation by increasing new neutrophil and platelet production. Studies on the neutrophil-lymphocyte ratio (NLR) and platelet-lymphocyte ratio (PLR) have shown their association with inflammatory diseases and are used as inflammation markers (12,13). There are also studies on the relationship of inflammation, which is a component of the pathophysiology of PCOS with NLR and PLR (14,15).

Cystatin C, hs-CRP, NLR, and PLR are markers in the literature related to inflammatory and metabolic processes (11,16). Therefore, we wanted to evaluate these parameters’ relationship with other metabolic parameters in normal weight and overweight adolescents with and without PCOS. This is the first study to evaluate these metabolic and inflammatory markers together in adolescent PCOS patients.

## MATERIALS AND METHODS

Ethical committee approval was received from the ethics committee of Amasya University on 3 January 2019 under protocol 3. Participants were selected from a group of patients 14-19 years old who presented to the gynecology outpatient clinics between January 3rd, 2019 and December 15th, 2019. All patients and their parents were informed about the study, and their consent was obtained. The World Health Organization (WHO) defines 10-19 years old as adolescence (17). Especially in the first two years with menarche, many adolescent girls experience menstrual irregularity due to anovulatory cycles (18). Therefore, PCOS diagnosis should be considered in clinical and/or biochemical hyperandrogenism along with oligomenorrhea after excluding other causes of menstrual irregularity and hyperandrogenism. Consequently, we tried to keep the age range between 14-19 years in our study (19). The average age of the groups was between 17 and 18 (Table 1). Adolescents who came to the clinic with menstrual irregularity complaints, hirsutism, and acne vulgaris were evaluated for the study group. Adolescents who met 3/3 of Rotterdam's PCOS criteria for adolescents (oligomenorrhea or chronic anovulation (at least two years after menarche), clinical or biochemical parameters of hyperandrogenism, and single ovarian volume greater than 10 mm^3^) were included in the study (20). Ninety adolescents included in the study were diagnosed with PCOS, but they had not received PCOS treatment for the last 6 months. There were also 100 adolescents who had regular menstrual cycles, no signs of hyperandrogenism, normal ovarian morphology ultrasonographically, and who visited the clinic for other complaints such as vaginal discharge or inguinal pain who constituted the control group. The study was described to the participants as a research study on PCOS, and almost all of the participants agreed to be included in the study.

**Table 1:** Demographic characteristics of the groups

		NW Control n (71)	OW Control n (27)	NW PCOS n (63)	OW PCOS n (26)	p
Age (year)		18 (14-19)	19 (15-19)	18 (14-19)	19 (15-19)	0.467
Age of menarche (year)		12 (9-13)	11 (10-13)	12 (10-13)	12 (11-13)	0.103
Height (cm)		160 (145-175)	161 (154-173)	165 (143-174)	162 (154-176)	0.110
Weight (cm)		58 (39-72)^a^	67 (60-75)^b^	60 (45-70)^a^	67 (60-91)^b^	**<0.001**
BMI (kg/m^2^)		22.77 (17.5-25.6)^a^	25.28 (24.6-28.8)^b^	23.05 (17.7-24.5)^a^	25.71 (24.5-34.3)^b^	**<0.001**
Education						
	Primary school	1 (1.4%)	0 (0%)	0 (0%)	0 (0%)	0.923
	Middle school	7 (9.9%)	3 (11.1%)	6 (9.5%)	3 (11.5%)	
	High School	48 (67.6%)	17 (63.0%)	39 (61.9%)	19 (73.1%)	
	University	15 (21.1%)	7 (25.9%)	18 (28.6%)	4 (15.4%)	
Previous operation		12 (16.9%)	5 (18.5%)	6 (9.5%)	2 (7.7%)	0.406
Chronic disease		4 (5.6%)	0 (0%)	3 (4.8%)	2 (7.7%)	0.585

p-values were calculated with the Kruskal Wallis and Chi-square test.

a, b There is no difference between groups with the same letters (p > 0.05). Different letters indicate significantly different groups (p < 0.05).

NW: normal weight; OW: overweight.

The Androgen Excess – PCOS Society Task Force suggests that the diagnosis of polycystic ovary morphology (PCOM) should be defined as ovarian follicles ≥ 20 with high-resolution transvaginal probes (21). PCOM is an inconsistent finding in adolescents where the ovaries appear to be enlarged and multi-follicular due to gonadotropin stimulation but a transvaginal probe is not suitable. PCOM is not associated with anovulation and metabolic pathologies (22). In this study, abdominal ultrasound was performed to evaluate the ovarian volume for PCOS and exclude ovarian pathologies that could cause hormonal and clinical disorders such as menstrual disorders. All ultrasonography was performed by the same experienced physician (A.T.T.). Therefore, it was implemented so that the inter-observer variability was minimal.

A Mindray DC-7 ultrasound device was used for ultrasound. Adolescents who were pregnant or who had diabetes mellitus, hyperprolactinemia, Cushing syndrome, congenital adrenal hyperplasia, adrenal, thyroid, hepatic, or renal or cardiovascular disease were excluded. We also excluded persons using hormone-containing drugs (e.g., oral contraceptives), statin group drugs, aspirin, non-steroidal anti-inflammatory drugs, or steroids. Patients were also questioned and examined for signs of infection. Those with signs of infection were excluded.

Participants in both groups were invited to the hospital on the second or third day of their menstrual period. (Those who did not menstruate for a long time due to oligomenorrhea were menstruated with medication.). Age, height, weight, waist and hip circumference, menstrual cycle patterns, and menstrual quantities of the participants were questioned; hirsutism was evaluated using the Ferriman-Gallwey score.

Blood samples were collected for fasting blood sugar, fasting insulin (to evaluate HOMA-IR), thyroid-stimulating hormone (TSH), prolactin (PRL), follicle-stimulating hormone (FSH), luteinizing hormone (LH), estradiol (E2), total testosterone, dehydroepiandrosterone sulfate (DHEAS), cholesterol, low-density lipid (LDL) cholesterol, high-density lipid (HDL) cholesterol, TG, neutrophils, lymphocytes, platelets, cystatin C, and hs-CRP levels. All adolescents were menstruating. Blood samples were taken on the 2nd or 3rd day of menstruation on an empty stomach in the morning between 8:00 and 8:30. These parameters were compared both between PCOS and control groups and for a normal versus overweight classification in order to evaluate the relationship between metabolic criteria of PCOS and BMI in adolescents. Not all adolescents with PCOS are overweight just as not all overweight adolescents have PCOS. BMI classification used age percentile curves for girls as recommended by the World Health Organization (23). Those with a percentile <5% were considered underweight, those 5% to <85% normal weight, those ≥85% to <95% overweight, and those ≥95% obese (24). At first, there were 90 adolescents in the PCOS group and 100 adolescents in the control group. Obese and underweight adolescents were excluded to avoid bias in metabolic parameters. Two adolescents in the control group were excluded from the study because they were <5% percentile, and one adolescent in the PCOS group was 95%. Thus, the groups were distributed into four subgroups: NW control (n = 71), OW control (n = 27), NW PCOS (n = 63), and OW PCOS (n = 26) (Table 2).

**Table 2:** Comparison of laboratory results, waist circumference, waist-hip ratio and Ferriman-Gallwey scores by classification of the groups as normal weight or overweight

	NW Control n (71)	OW Control n (27)	NW PCOS n (63)	OW PCOS n (26)	p
Cystatin C (mg/L)	0.65 (0.45-0.74)^a^	0.66 (0.5-0.74)^a^	0.75 (0.65-0.88)^b^	0.75 (0.64-0.9)^b^	**<0.001**
Hs-CRP (mg/L)	2 (0.14-5.76)^a^	2.45 (0.23-5.04)^a^	4.3 (3.31-6.22)^b^	4.2 (3.03-5.67)^b^	**<0.001**
NLR	1.75 (0.6-7.63)^a^	1.73 (0.61-3.89)^a^	2.41 (1.25-25.13)^b^	1.97 (0.79-6.01)a,b	**<0.002**
PLR	130.43 (46.71-292.22)	118.9 (69.05-236.07)	127 (56.93-621.43)	118.33 (57.46-304.58)	0.101
C (mg/dL)	156.17 ± 20.67	164.25 ± 16.71	166.00 ± 25.98	165.07 ± 49.62	0.184^VA^
LDL (mg/dL)	100.73 ± 19.35	104.07 ± 22.50	104.96 ± 24.86	109.76 ± 31.66	0.395
HDL (mg/dL)	54.53 ± 8.64	59.66 ± 11.18	54.57 ± 10.89	54.95 ± 15.94	0.184
TG (mg/dL)	84 (35-238)^a^	85 (39-187)^a^	110 (37-252)^b^	145 (25-222)^b^	**<0.001**
FBG (mg/dL)	78 (70-118)^a^	84 (70-135)b,c	83 (74-117)^b^	94 (72-128)^c^	**<0.001**
FI (mIU/L)	7.15 (3.84-82.03)^a^	8.83 (3.86-13.16)a,b	10.23 (7.02-14.35)b,c	11.72 (8.89-15.54)^c^	**<0.017** VA
HOMA-IR	1.4 (0.7-2.4)^a^	1.8 (0.8-2.8)^b^	2.2 (1.3-3.1)^b^	2.9 (1.6-4.1)^c^	**<0.001**
FSH (mIU/L)	6.15 ± 1.52	6.27 ± 1.19	6.26 ± 1.61	6.19 ± 1.79	0.973
LH (mIU/L)	5.47 (1.74-10.8)^a^	6.47 (2.47-9.8)^a^	15.31 (6.11-34)^b^	14.5 (4.94-26.1)^b^	**<0.001**
E2 (pg/mL)	38.7 (9.16-106)^c^	34.91 (18.9-109)b,c	42.8 (12.3-223)a,b	48.7 (16-257)^a^	**<0.001**
T (ng/dL)	37 (6.9-68)^c^	39 (17.6-76)c,b	43.9 (16-132)a,b	56 (18.9-120)^a^	**<0.001**
DHEAS (IU/mL)	223 (125-412)^a^	224 (98-409)^a^	289 (104-729)^b^	299 (148-644)^b^	**<0.001**
TSH (mIU/L)	1.96 ± 1.03	2.11 ± 0.98	1.89 ± 0.88	2.19 ± 1.04	0.524
WC (cm)	72 (64-79)^a^	73 (65-77)^a^	76 (68-80)^b^	76 (68-79)^b^	**<0.001**
WHR (cm/cm)	0.78 (0.68-0.91)^a^	0.79 (0.69-0.89)^a^	0.85 (0.67-0.93)^b^	0.84 (0.75-0.93)^b^	**<0.001**
FGS	5 (2-8)^a^	5 (3-9)^a^	9 (5-18)^b^	9 (6-19)^b^	**<0.001**

p-values were calculated with the Kruskal Wallis test and One-way analysis of variance test.

a,b,c There is no difference between groups with the same letters (p > 0.05). Different letters indicate significantly different groups (p < 0.05).

VA One-way analysis of variance test statistic. NW: normal weight; OW: overweight; NLR: neutrophil lymphocyte ratio; PLR: platelet lymphocyte ratio; C: cholesterol; FBG: fasting blood glucose; FI: fasting insulin; TT: total testosterone; WC: waist circumference, WHR: waist-hip ratio; FGS: Ferriman-Gallwey score.

TSH, PRL, FSH, LH, E2, total testosterone, and DHEAS were studied using an electrochemiluminescence immunoassay (Advia Centaur XP, Siemens, Germany); cholesterol, LDL, HDL, and TG were studied using enzymatic colorimetric tests (Cobas C 702, Roche, Japanese); and neutrophils, lymphocytes, and platelets were studied using laser optics (X N-1000, Siemens, Japan). Hs-CRP was studied using an immunoturbidimetric test (Cobas C 702, Roche, Japanese), and cystatin C was evaluated using immunonephelometric tests (Dade Behring, Germany). Fasting blood glucose (Cobas C 702, Roche, Japanese) and fasting insulin (Advia Centaur XP, Siemens, Germany) were measured using electrochemiluminescence. Homeostatic model assessment insulin resistance index (HOMA-IR) was calculated using fasting blood glucose (mg/dL) * fasting insulin (mIU/L) / 405.

### Power analysis

Sample size was calculated by a statistician who performed the analysis using the G * Power 3.1 program considering Yılmaz and cols. (14) along with effect size (w = 0.951) and double-tailed hypothesis method. The confidence interval was determined to be 95%, and the margin of error was 5%. According to the calculation, there should be 86 women in the control group and 86 women in the experimental group for 174 women in total.

### Statistical analysis

Data were analyzed with IBM SPSS V23 program. Conformity to normal distribution was examined with Kolmogorov-Smirnov test. Chi-square test and Fisher's Exact test were used to compare categorical variables according to groups. The Mann-Whitney U test was used for comparing non-normally distributed data according to paired groups and independent two sample t test was used for comparison of normally distributed data. One-way analysis of variance (ANOVA) was used to compare normally distributed data for three or more groups, and the Kruskal Wallis test was used for non-normally distributed data. Results are presented as mean ± standard deviation and median (minimum-maximum) for quantitative data and as frequency (percentage) for categorical data. The significance level was p < 0.05. Binary logistic regression analysis was used to examine the risk factors affecting PCOS. Spearman's rho correlation coefficient was used to examine the relationship between non-normally distributed quantitative variables. ROC (Receiver operating curve) analysis was used to determine cut-off values for cystatin C, hs-CRP, and NLR for PCOS groups. The significance level was p < 0.05.

## RESULTS

Groups were homogeneous in demographic characteristics (Table 1). Normal vs. overweight subgroups comparison is shown in Table 2. There was no significant difference in BMI between NW control and NW PCOS or between OW control and OW PCOS. The groups had a homogeneous distribution.

Mean cystatin C, hs-CRP, TG, LH, DHEAS, waist measurement, WHR, and FGS values were significantly higher in adolescents for both NW and OW PCOS compared to the control groups (p < 0.05).

NLR was highest in the NW PCOS group. While there was no significant difference from OW PCOS (p > 0.05), and it was significant versus controls (p < 0.05). There was no significant difference between OW PCOS and controls (p > 0.05).

The mean FBG value in NW PCOS patients was significantly higher than NW controls (p < 0.05), but not different from the OW controls (p > 0.05). However, it was highest in the OW PCOS group, and there was no significant difference from the NW PCOS group; it was significantly higher than both NW and OW controls (p < 0.05).

Fasting insulin was highest in the OW PCOS group while there was no significant difference versus NW PCOS (p > 0.05). It was significantly different from controls (p < 0.05). There was no significant difference versus OW controls in NW PCOS (p > 0.05), but it was significant versus NW controls (p < 0.05).

While HOMA-IR values had no significant difference between the NW PCOS and OW controls, these two groups were significantly higher than NW controls. This value was highest for OW PCOS patients and this was statistically significant versus the other three groups (p < 0.05).

The E2 was highest in the OW PCOS group. There was no significant difference from NW PCOS (p > 0.05), but there was a significant difference versus controls (p < 0.05). There was no significant difference versus OW controls in NW PCOS (p > 0.05), but the difference from NW controls was significant (p < 0.05).

Total testosterone levels were highest in the OW PCOS group. There was no significant difference between NW PCOS and OW PCOS. However, testosterone levels in OW PCOS were significantly higher than in NW controls and OW controls (p < 0.05).

**Figure 1:**
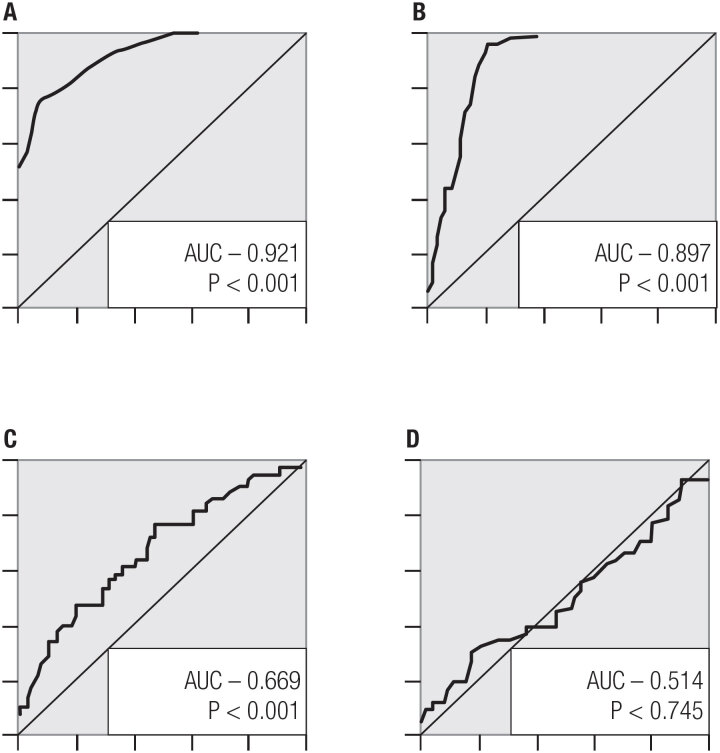
(**A**) ROC curve for cystatin C; (**B**) ROC curve for Hs-CRP; (**C**) ROC curve for NLR; (**D**) ROC curve for PLR.

Cystatin C, hs-CRP, NLR, and PLR were evaluated with receiver operator characteristics curve (ROC). The area under the curve (AUC) in ROC of cystatin C, hs-CRP, and NLR was statistically significant (p < 0.05). In the ROC analysis, cystatin C's sensitivity and specificity were 78.9 and 84.2, hs-CRP's 82.6 and 85.7, and PLR's 58.5 and 63.5 respectively (p < 0.05). This is shown in Table 3 and Figure 1.

**Table 3:** Sensitivity, specificity, positive predictive and negative predictive values for cystatin C, hs-CRP, NLR, and PLR

	Area	P	Cutt-off	Lower	Upper	Sensitivity	Specificity	PPV	NPV
**Cystatin C**	0.921	**<0.001**	0.685	0.884	0.956	78.9	84.2	83.3	80
**Hs-CRP**	0.897	**<0.001**	3.595	0.849	0.945	82.6	85.7	84.4	84
**NLR**	0.669	**<0.001**	1.906	0.592	0.745	58.5	63.5	61.1	61
**PLR**	0.514	0.745	126.84	0.431	0.597	49.5	54.7	52.2	52

*p* values were calculated according to ROC analysis. PPV: positive predictive value; NPV: negative predictive value.

Regression analysis was performed to assess these parameters’ association with PCOS better, as shown in Table 4. The univariate regression analysis showed that the risk of PCOS increased 1.556 times when cystatin C increased by one unit, the risk of PCOS increased 5.815 times when hs-CRP increased by one unit, and the risk of PCOS increased 1.561 times when NLR increased by one unit (p < 0.001, p < 0.001 and p = 0.003; respectively). There was an increased risk of PCOS with the increase of TG, FBG, FI, HOMA-IR, LH, E2, TT, DHEAS, WC, WHR, and FGS (p < 0.05).

**Table 4:** Examination of risk factors affecting PCOS by binary logistic regression

	Univariate		Multivariate Model 1		Multivariate Model 2	
	OR (%95 CI)	p	OR (%95 CI)	p	OR (%95 CI)	p
BMI (kg/m^2^)	1.064 (0.941-1.205)	0.322				
Cystatin C (mg/L)	1.556 (1.36-1.781)	**<0.001**			1.546 (1.282- 1.864)	**<0.001**
Hs-CRP (mg/L)	5.815 (3.448-9.808)	**<0.001**			4.092 (2.201- 7.608)	**<0.001**
NLR	1.561 (1.162-2.096)	**0.003**			1.299 (0.793- 2.13)	0.299
PLR	1.004 (0.999-1.008)	0.098			0.999 (0.987- 1.012)	0.904
C (mg/dL)	1.01 (0.999-1.021)	0.078				
LDL (mg/dL)	1.009 (0.996-1.021)	0.175				
HDL (mg/dL)	0.99 (0.964-1.016)	0.435				
TG (mg/dL)	1.019 (1.011-1.027)	**<0.001**				
FBG (mg/dL)	1.055 (1.029-1.083)	**<0.001**				
FI (mIU/L)	1.334 (1.167-1.526)	**<0.001**				
HOMA-IR	26.136 (10.437-65.45)	**<0.001**				
FSH (mIU/L)	1.026 (0.851-1.237)	0.785	0.193 (0.066-0.568)	**0.003**		
LH (mIU/L)	3.294 (2.14-5.071)	**<0.001**	7.852 (2.528-24.389)	**<0.001**		
E2 (pg/mL)	1.027 (1.01-1.044)	**0.001**				
TT (ng/dL)	1.042 (1.023-1.062)	**<0.001**				
DHEAS (IU/mL)	1.011 (1.007-1.016)	**<0.001**				
WC (cm)	1.405 (1.258-1.57)	**<0.001**				
WHR (cm/cm)	1.17 (1.104-1.239)	**<0.001**	1.671 (1.1-2.539)	**0.016**		
FGS	4.228 (2.741-6.523)	**<0.001**				

Multivariate Model 1: Backward: Wald method was used to include independent risk factors in the model. Model 2: Enter method was used to include independent risk factors in the model.

NLR: neutrophil lymphocyte ratio; PLR: platelet lymphocyte ratio; C: cholesterol; FBG: fasting blood glucose; FI: fasting insulin; TT: total testosterone; WC: waist circumference; WHR: waist-hip ratio; FGS: Ferriman-Gallwey score.

In multivariate regression analysis, in model 2, only 4 parameters (Cystatin c, hs-CRP, PLR, and NLR) were included in the model, while in model 1, all parameters in the univariate part were included in the model. According to the results of the multivariate model 1, the risk of PCOS increases 0.193 times (p = 0.003) as FSH increases, 7.852 times (p < 0.001) as LH increases, and 1.671 times (p = 0.016) as WHR increases. According to Model 2 results, the risk of PCOS increases 1.546 times (p < 0.001) as cystatin C increases and 4.092 times as hs-CRP increases (p<0.001). According to multivariate model 2, NLR and PLR were not significant (p > 0.05).

The relationship between cystatin C, hs-CRP, NLR and PLR with BMI, serum lipids, HOMA-IR, FBG, FI, WHR, and WC are shown in Table 5. Cystatin C correlated with cholesterol, TG, HOMA-IR, FBG, WHR and WC. Hc-CRP levels correlated with TG, HOMA-IR, FBG and WC. Only HOMA-IR correlated with NLR. None of these parameters correlated with PLR.

**Table 5:** Relationship of cystatin C, hs-CRP, NLR and PLR with BMI, serum lipid, HOMA-IR, FBG, FI, WHR and WC

		Cystatin C (mg/L)	Hs-CRP (mg/L)	NLR	PLR
BMI (kg/m^2^)	r	0.138	0.063	-0.049	-0.030
	p	0.059	0.389	0.509	0.688
Cholesterol (mg/dL)	r	0.195	0.130	0.101	0.063
	p	**0.008**	0.076	0.169	0.392
LDL (mg/dL)	r	0.113	0.103	0.054	0.032
	p	0.123	0.161	0.462	0.664
HDL (mg/dL)	r	-0.029	-0.030	0.045	0.122
	p	0.696	0.682	0.537	0.096
TG (mg/dL)	r	0.328	0.293	0.140	0.096
	p	**<0.001**	**<0.001**	0.056	0.190
HOMA-IR	r	0.479	0.411	0.163	0.091
	p	**<0.001**	**<0.001**	**0.026**	0.214
FBG (mg/dL)	r	0.287	0.239	0.032	-0.002
	p	**<0.001**	**0.001**	0.663	0.974
FI (mIU/L)	r	0.072	0.090	0.094	0.060
	p	0.330	0.219	0.203	0.417
WHR (cm/cm)	r	0.273	0.334	0.070	-0.056
	p	**<0.001**	**<0.001**	0.340	0.450
WC (cm)	r	0.357	0.379	0.066	-0.048
	p	**<0.001**	**<0.001**	0.368	0.517

p-values were calculated with the Spearman's rho correlation coefficient test. r: Spearman's rho correlation coefficient. NLR: neutrophil lymphocyte ratio; PLR: platelet lymphocyte ratio; FBG: fasting blood glucose; FI: fasting insulin; WHR: waist-hip ratio; WC: waist circumference.

## DISCUSSION

PCOS is a common factor of insulin-resistant hyperinsulinism partially associated with obesity (25). Obesity and insulin resistance are frequently seen in PCOS and also trigger chronic inflammation (7). In our study, cystatin C, hs-CRP, NLR, triglyceride, FBG-FI, HOMA-IR, WC, and WHR were significantly higher in those with PCOS. Even if the PCOS group adolescents were NW, they had significantly higher TG, cystatin C, hs-CRP, and NLR than OW controls. The highest HOMA-IR values were observed in OW PCOS (p < 0.05). Cystatin C and hs-CRP sensitivity and specificity were significant (p < 0.05). Cystatin C and hs-CRP were positively correlated with other metabolic parameters. In addition, an increase in the risk of PCOS was observed with an increase in cystatin C, hs-CRP by one unit (Table 4).

The International Diabetes Federation defined the criteria for metabolic syndrome in adolescents. In addition to central obesity (assessed by waist circumference), the presence of two symptoms among high fasting blood glucose, high systolic or diastolic blood pressure, or high TG or low HDL meets the diagnosis of metabolic syndrome (26). Waist circumference, FBG, TG, and HDL were evaluated in this study. FBG, TG and WC were high in adolescents with PCOS. In this study, the same values were higher in both NW and OW PCOS compared to controls except for FBG. The fact that FBG was significantly higher in the group with OW PCOS and the parameters indicating insulin resistance, HOMA-IR, and FI were higher in the NW and OW PCOS groups especially in the NW PCOS group indicates the risk of metabolic syndrome in adolescents with PCOS independent of BMI.

One of the 5 criteria suggested by Sultan and Paris in the diagnostic evaluation of PCOS in adolescents is insulin resistance or hyperinsulinism (27). In our study, OW adolescents in the control group had similar FI and HOMA-IR levels even though adolescents with PCOS were NW. The highest HOMAIR level was found to be significant in the OW PCOS group. This situation supports insulin resistance in adolescents with PCOS independent of BMI but also suggests that weight gain affects parameters more negatively.

Some studies have shown that cystatin C is important in diagnosing metabolic syndrome and determining risk (8,28). In our study, when we divided groups into NW and OW according to their BMI for age percentile curves for adolescent girls, cystatin C was higher in both PCOS groups versus healthy adolescents (p < 0.05) (Table 2). In addition, cardiometabolic risk factors such as HOMA-IR, TG, waist circumference, and WHR were higher in OW PCOS compared to the OW controls although there was no difference in BMI. This situation supports findings of increased cardiometabolic risk in PCOS. Similar to our results, Çınar and cols. found cystatin C levels to be higher in adolescents with PCOS suggesting that cystatin C is a promising marker in establishing PCOS in adolescents (10). Gozashti and cols. also found that cystatin C values were higher in patients with PCOS (29). In another study, patients with PCOS were grouped as those with and without metabolic syndrome, and cystatin C correlated with LDL, cholesterol, TG, and total cholesterol (8). High TG and cystatin C levels in our study also support this result. In our study, cystatin C and hs-CRP levels correlated with cholesterol, TG, HOMA-IR, FBG, WHR, and WC.

Studies have shown a relationship between hs-CRP and inflammatory processes such as atherosclerosis and type 2 diabetes mellitus (11,30). We found that adolescents with PCOS, whether normal weight or overweight, had higher hs-CRP than controls (p < 0.05) (Table 2). In three different studies without any obesity distinction, hs-CRP was significantly higher in PCOS similar to our results (10,29,31). In a study where hs-CRP and lipid parameters were evaluated in non-obese adolescent girls, hs-CRP levels were significantly higher in adolescents with PCOS and correlated with cholesterol and LDL (32). Ün and cols. reported no difference between a control group and patients with PCOS in hs-CRP, but hs-CRP values were significantly higher in patients with obesity when patients with PCOS were considered as obese/non-obese (33). In our study, groups were stratified as NW and OW because the number of obese adolescents was low and to eliminate bias that may occur due to obesity and underweight.

We also wanted to evaluate NLR and PLR in PCOS because inflammation plays a role in its pathophysiology. In our study, NLR was higher in adolescents with PCOS compared to controls (p < 0.05). However, PLR values did not differ significantly for both groups of PCOS compared to controls (p > 0.05) (Table 2). In the study of Çakıroğlu and cols., both NLR and PLR were high in patients with PCOS. Contrary to our conclusion, PLR was significantly elevated in normal-weight patients with PCOS (34). Pergialiotis and cols. found that NLR and PLR were not affected by the presence of obesity (15). Yılmaz and cols. found that, similar to hs-CRP levels, NLR levels were also higher in obese and lean PCOS patients compared to controls. However, these values did not differ significantly in obese PCOS patients similar to our study (14). All of these results with elevated inflammation markers (cystatin C, hs-CRP, NLR) in both NW and OW PCOS indicate that PCOS is formed depending on the inflammatory process and is independent of obesity. Keskin and cols. showed that inflammatory markers are increased in the PCOS group in adults; differently, our study shows that the metabolic and inflammatory process starts in adolescence (35).

In our study, blood pressure monitoring which is included in the definition of metabolic syndrome in children and adolescents by the International Diabetes Federation was not available. Waist circumference, TG, HDL and FBG values were available (26). The absence of blood pressure measurements is one of the limitations of our study. Adolescents with obese PCOS could not be evaluated because we did not have obese PCOS cases (there was only one case) that we could include in our study. If obese cases could be included and especially if they could be matched with an obese control group then the relationship between metabolic syndrome risk in adolescents with PCOS and BMI could be better demonstrated. We evaluated metabolic syndrome criteria except for high blood pressure, but the values obtained were not within the pathological values that the International Diabetes Federation specified as the lower limit; this is a limitation (26). We evaluated the significance of parameters in the study not according to pathological levels but according to their significantly higher values than controls. Since most of the adolescents diagnosed with PCOS could not menstruate for a long time due to oligomenorrhea, we gave them medication to ensure their menstruation. And only in this way the laboratory examinations required for the study could be carried out, which is another limitation of our study.

Our study's strengths are that the groups were matched in terms of demographic characteristics and especially BMI. This is the first study to compare all these values together, which is a major strength. We also compare inflammatory and metabolic parameters between the groups such as WC and WHR and then evaluate the correlations of serum parameters with each other (Table 5). Moreover, we tried to show the risk factors for PCOS and how much they increase PCOS risk (Table 4).

In summary, the homogeneity of BMI distribution between the NW controls and the NW PCOS group and between the OW controls and the OW PCOS group gave us a match in terms of weight. In this way, cystatin C, hs-CRP, and NLR, which we can call inflammation markers, are higher in adolescents with both NW and OW PCOS than controls, supporting that PCOS develops in the inflammatory process, independent of obesity. Also, waist circumference, WHR, FBG, FI, HOMA-IR, and TG, which are cardiometabolic risk markers, regardless of their weight, were higher in adolescents with PCOS than the controls (especially in the OW control group). It also supports that PCOS has a metabolic syndrome component. The results obtained in our study pave the way for the use of cystatin C and hs-CRP values appear to be promising markers in the follow-up of adolescents with PCOS and predicting long-term cardiometabolic risks.
